# Supplementary material for: Synthesis of Electron-Rich Porous Organic Polymers via Schiff-Base Chemistry for Efficient Iodine Capture
Source: Molecules. 2022 Aug 12;27(16):5161. doi: 10.3390/molecules27165161 (PMC9415008; doi:10.3390/molecules27165161)
Supplement: Supplementary file 1 [file molecules-27-05161-s001.zip › molecules-1850469-supplementary.pdf]

Supplementary Materials

Synthesis of Electron-Rich Porous Organic Polymers via Schiff-Base Chemistry for Efficient Iodine Capture

Peng Tian [1,†], Zhiting Ai [1,†], Hui Hu [1], Ming Wang [1], Yaling Li [1], Xinpei Gao [1], Jiaying Qian [1], Xiaofang Su [1,*], Songtao Xiao [2,*], Huanjun Xu [3], Fei Lu [1] and Yanan Gao [1,*]

1 Key Laboratory of Ministry of Education for Advanced Materials in Tropical Island Resources, Department of Chemistry and Chemical Engineering, Hainan University, No 58, Renmin Avenue, Haikou 570228, China

2 China Institute of Atomic Energy, Beijing 102413, China

3 School of Science, Qiongtai Normal University, Haikou 571127, China

* Correspondence: sxf@hainanu.edu.cn (X.S.); xiao200112@163.com (S.X.); ygao@hainanu.edu.cn (Y.G.)

† Those authors contribute to this work.

Table of Contents

Section 1. Materials and instruments

Section 2. Synthetic procedures

Section 3. Iodine adsorption and desorption procedures

Section 4. Characterization of POPs

Section 5. Iodine vapor adsorption and desorption

Section 6. Iodine solution adsorption experiment

Section 1. Materials and instruments

Materials

All the chemical reagents were purchased from commercial sources and used without further purification. Anhydrous 1,3,6,8-tetrabromopyrene (98%) and 4,4',4'',4'''-([2,2'-bi(1,3-dithiolylidene)]-4,4',5,5'-tetrayl)tetraaniline (TTF-4NH_2) were obtained from EXTENSION Jilin, China. 1-Bromoadamantane (98%), tert-butyl bromide anhydrous (98%), aluminum chloride (98%), benzophenone (98%) and 4-aminophenylboronic acid pinacol ester (98%) were purchased from MERYER Shanghai, China. Tetrahydrofuran (99%), 1,4-dioxane (99%), zinc powder (98%), titanium tetrachloride (98%) and 1,1-dichlorodimethyl ether were purchased from Innochem Beijing, China. Acetic acid (99%) and 1,2-dichlorobenzene (99%) were purchased from TCI, Japan. Cesium carbonate (99%), magnesium sulphate (99%) and tetrakis(triphenylphosphine)palladium (99%) were purchased from Tansoole Shanghai, China.

Methods

Power X-ray diffraction (PXRD) data were recorded on a Rigaku X-ray diffractometer XRD-MiniFlex600 (Cu-Kα radiation; λ=1.542Å). The sample is dried under vacuum and then mounted on a glass flat sample plate. Patterns were collected in the 2º<2θ<30º range with a step size of 0.02º and exposure time of 10º/min. Fourier transform infrared (FT-IR) spectra were recorded in a Jasco FT/IR-6800 accessory with a spectral range of 4000-500 cm^{-1}, signals are

given in wavenumbers (cm^{-1}). Thermogravimetric analyses (TGA) were run on a Thermbalance TGA Q600 thermal gravimetric analyser with samples under N_2 atmosphere. The samples were heated at 10 °C min^{-1} within a temperature range of 25-900 °C. N_2 adsorption and desorption isotherms (BET) were done at 77K using Quantachrome Autosorb-iQ. Before the test, the sample was vacuum dried overnight at 120 °C. By using the non-local density functional theory (NLDFT) model, the pore volume was derived from the sorption curve. Scanning Electron Microscopy (SEM) studies were performed on a thermoscientific Verios G4 UC. The sample was dispersed in ethanol, and dropped on a piece of clean conductive tape, coated with gold. Ultraviolet visible infrared spectrophotometer (UV-vis) spectra were recorded in a Jasco V-770 accessory with a spectral range of 200-700 nm, signals are given in wavenumbers (nm). ^1H NMR was tested with a 400M NMR spectrometer (Bruker AVANCE NEO 400MHz NMR spectrometer).

Section 2. Synthetic procedures

Synthesis of 1,3,5,7-tetrakis (4-formylphenyl) adamantane (TFPA)

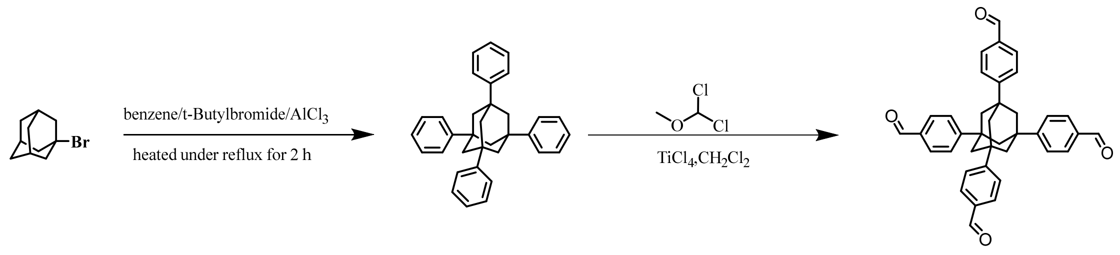

Figure S1. Scheme for the synthesis of TFPA monomer.

(1) Synthesis of 1,3,5,7-tetraphenyladamantane (TPA)

TPA was first synthesized according to a modified method.[1] 1-Bromoadamantane (46.5 mmol, 10.0 g) was added into a 500 mL three-necked round bottom flask and the flask was degassed by freeze-pump-thaw cycles and purged with nitrogen for three times, and then 200 mL anhydrous benzene was added into the flask and stirred to dissolve 1-bromoadamantane completely. After that, anhydrous $AlCl_3$ (2.0 g, 15 mmol) and tert-butyl bromide (10.5 mL, 93 mmol) were added into to the flask and refluxed for 6 hours at 75 °C. After cooling down to room temperature, the mixture was poured into cold water, and ether was then added under vigorously stirring condition. The solid was filtered off and washed with chloroform (250 mL) and ethanol (250 mL), successively. The product was dried under reduced pressure. The crude product was directly used for the next reaction without further purification (16.9 g, 38.4 mmol, 82% in yield).

(2) Synthesis of TFPA

TFPA was first synthesized according to a modified method.[2] TPA (3.8 g, 8.6 mmol) was added into a 250 mL three-necked round bottom flask. The flask was degassed by freeze-pump-thaw cycles and purged with nitrogen for three times. 80 mL anhydrous dichloromethane was added into the flask, and the flask was then cooled to -10 °C. After that, $TiCl_4$ (19 mL, 172.4 mmol) was slowly added to the mixture in the flask, and stirred for 1 hour. Finally, 1,1-dichlorodimethyl ether was added dropwise to the flask. The reaction was held at -15 °C for 3 hours and then allowed to warm up to room temperature with stirring overnight. The mixture was poured into 400 mL of ice water, then 2 M HCl (100 mL) was added, and the hydrolysis reaction was carried out for 1 hour. The two-phase mixture was separated and the aqueous phase was washed twice with 200 mL dichloromethane. The combined organic phases were washed with 1M HCl, deionized water, saturated aqueous $NaHCO_3$ and saturated aqueous NaCl. The crude product was obtained after vacuum distillation. The crude product was purified by column chromatography with dichloromethane and then recrystallized from dioxane to give crystals of TFPA as a white powder (0.9 g, 18% in yield). ^1H NMR (400 MHz, $CDCl_3$) δ 10.02 (s, 4H), 7.91 (s, 8H), 7.68 (s, 8H), 2.27 (s, 12H). (see Fig S2)

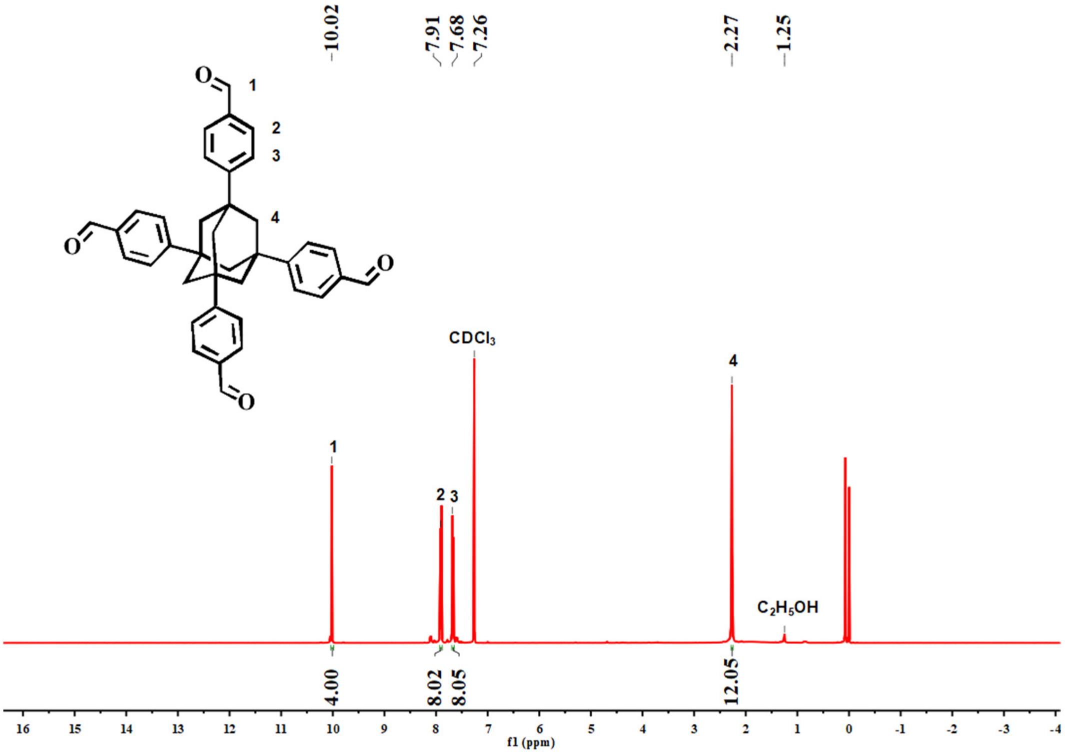

Figure S2. ^{1}H NMR spectrum of TFPA.

Synthesis of Tetrakis (4-aminophenyl) ethene (TAPE)

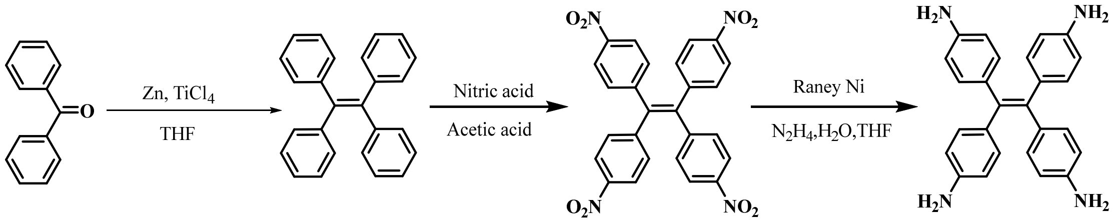

Figure S3. Scheme for the synthesis of TAPE monomer.

(1) Synthesis of tetraphenylethylene (TPE)

TPE was first synthesized according to a modified method.[3] Zinc (26.0 g, 400 mmol) was added into a three-necked round bottom flask, which was degassed by freeze-pump-thaw cycles and purged with nitrogen for three times. 50 mL of anhydrous THF was then added into the flask and refluxed for 2 hours at 75 °C. A solution of benzophenone (18.2 g, 100 mmol) in dry THF (100 mL) was added slowly to the flask and refluxed overnight. After cooling down to room temperature, the reaction mixture was quenched with saturated NaCl aqueous solution and extracted with deionized water and dichloromethane. The organic phase was retained, dried over anhydrous magnesium sulfate, filtered and distilled under reduced pressure. The product was dried under reduced pressure (14.4 g, 88% in yield). The crude product was directly used for the next reaction without further purification.

(2) Synthesis of Tetrakis (4-nitrophenyl) ethene (TNPE)

TNPE was synthesized according to a modified method.[3] Concentrated sulfuric acid (16 mL) and fuming nitric acid (28 mL) were added to a round-bottomed flask under ice bath condition. The mixture was further cooled down to -40 °C, stirred for 20 min to fully mix the mixture. TPE (2.0 g) was then added into the mixture in batches. The reaction was returned to room temperature and kept for 24 hours under stirring. After that, the mixture was poured into 300 mL of ice water and solids were collected by filtration, recrystallized from dioxane, and dried in vacuo to give crystals of TAPE as a yellow powder (3.0 g, 50% in yield). ^{1}H NMR (400 MHz, $CDCl_3$) δ 8.06 (d, J = 8.8 Hz, 8H), 7.19 (d, J = 8.8 Hz, 8H). (see Fig S4)

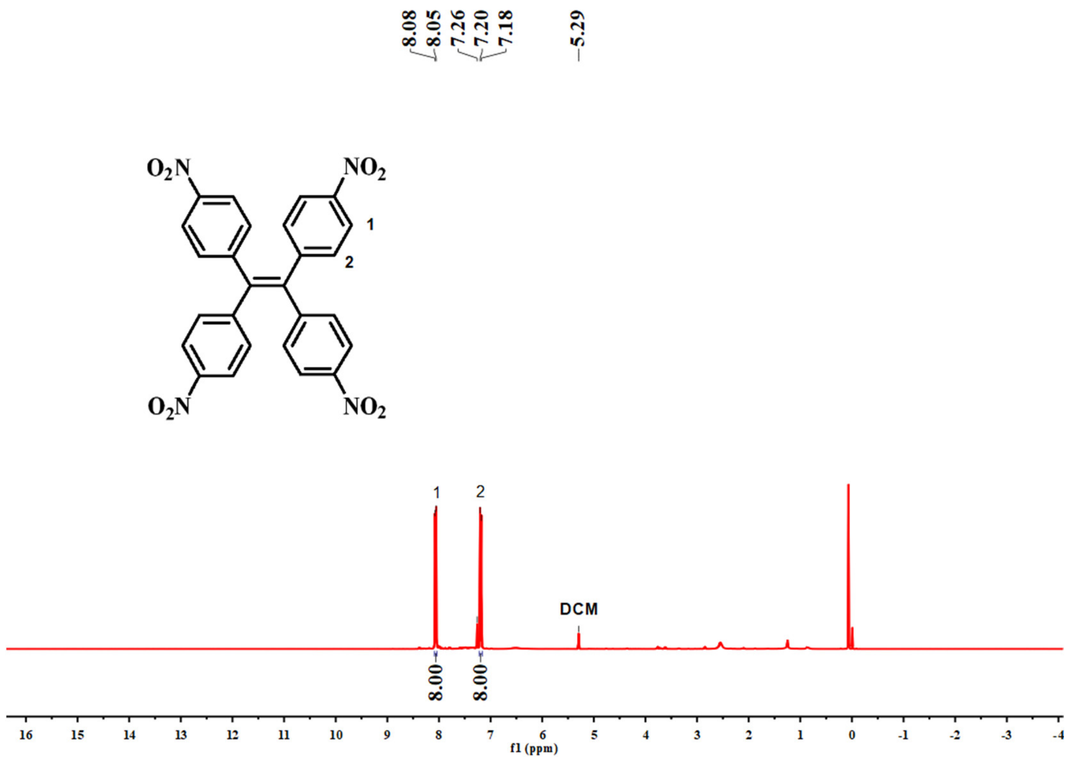

Figure S4. ^{1}H NMR spectrum of TNPE.

(3) Synthesis of TAPE

TAPE was synthesized according to a modified method.[3] TNPE (0.53 g, 1.04 mmol) and tetrahydrofuran (20 mL) were added to a round-bottomed flask. After TNPE was completely dissolved, Raney nickel (2.0 g, 34.0 mmol) was added. Then, hydrazine hydrate (0.67 mL) was slowly added to the flask and refluxed for 2 hours at 70 °C. The mixture was washed with tetrahydrofuran and the filtrate was filtered using diatomaceous earth. The TAPE product was dried under reduced pressure (0.4 g, 80% in yield). ^{1}H NMR (400 MHz, DMSO) δ 6.56 (s, 8H), 6.27 (s, 8H), 4.84 (s, 8H). (see Fig S5)

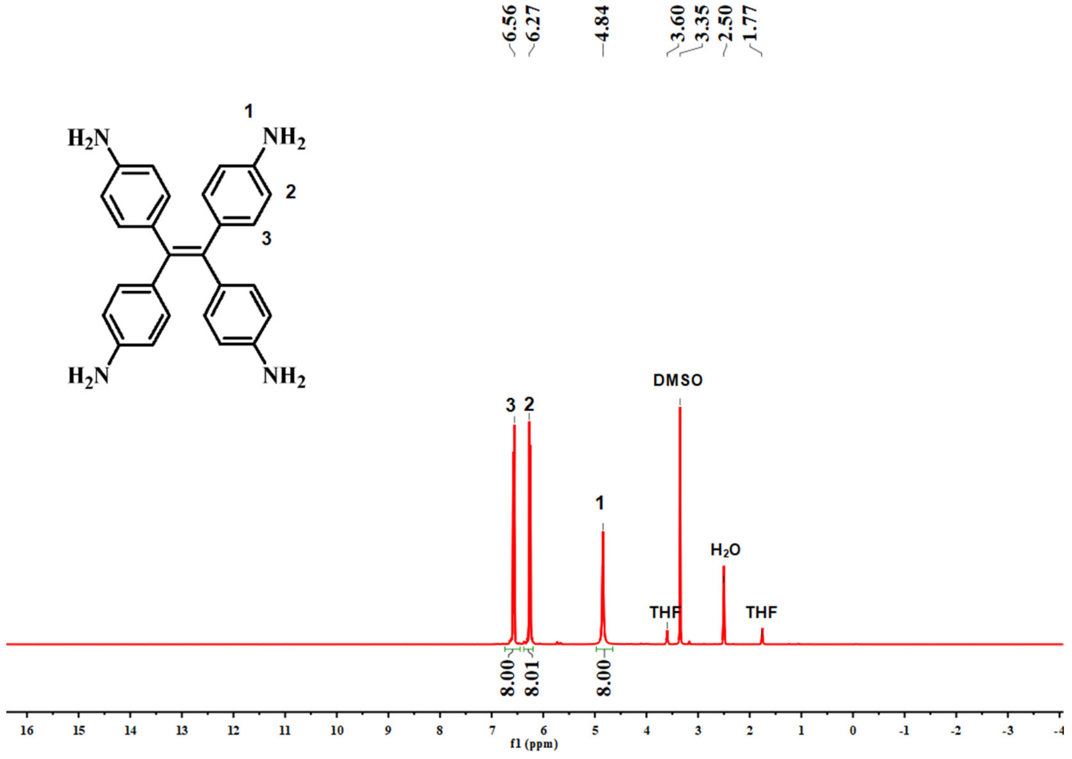

Figure S5. ^{1}H NMR spectrum of TAPE.

Synthesis of 1,3,6,8-tetrakis (4-aminophenyl) pyrene (PyTTA)

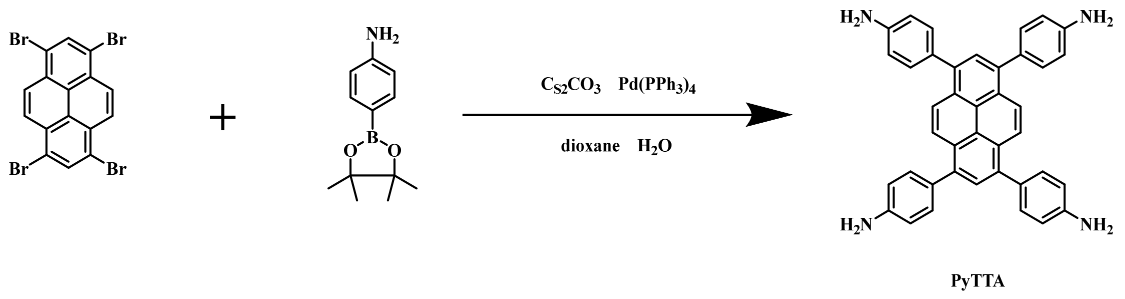

Figure S6. Scheme for the synthesis of PyTTA monomer.

PyTTA was first synthesized according to a modified method.[4] Typically, 1,3,6,8-tetrabromopyrene (0.74 g, 1.43 mmol), 4-aminophenylboronic acid pinacol ester (1.32 g, 6.02 mmol), Cesium carbonate (2.61g, 8.01 mmol) and $Pd(PPh_3)_4$ (0.17 g, 0.15 mmol) were added into a 250 mL three-necked round bottom flask, degassed by freeze-pump-thaw cycles and purged with nitrogen for three times, then dioxane and water were added into the flask and refluxed for 72 hours at 115 °C. The mixture was washed with plenty of water until the pH of the filtrate showed neutral and crude product was obtained after vacuum distillation. The crude product was purified by column chromatography with (ether/ethyl acetate =1:1) to afford bright yellow PyTTA (0.6 g, 70% in yield). ^1H NMR (400 MHz, DMSO) δ 8.14 (s, 4H), 7.80 (s, 2H), 7.35 (d, J = 8.3 Hz, 8H), 6.78 (d, J = 8.4 Hz, 8H), 5.32 (s, 8H). (see Fig S7)

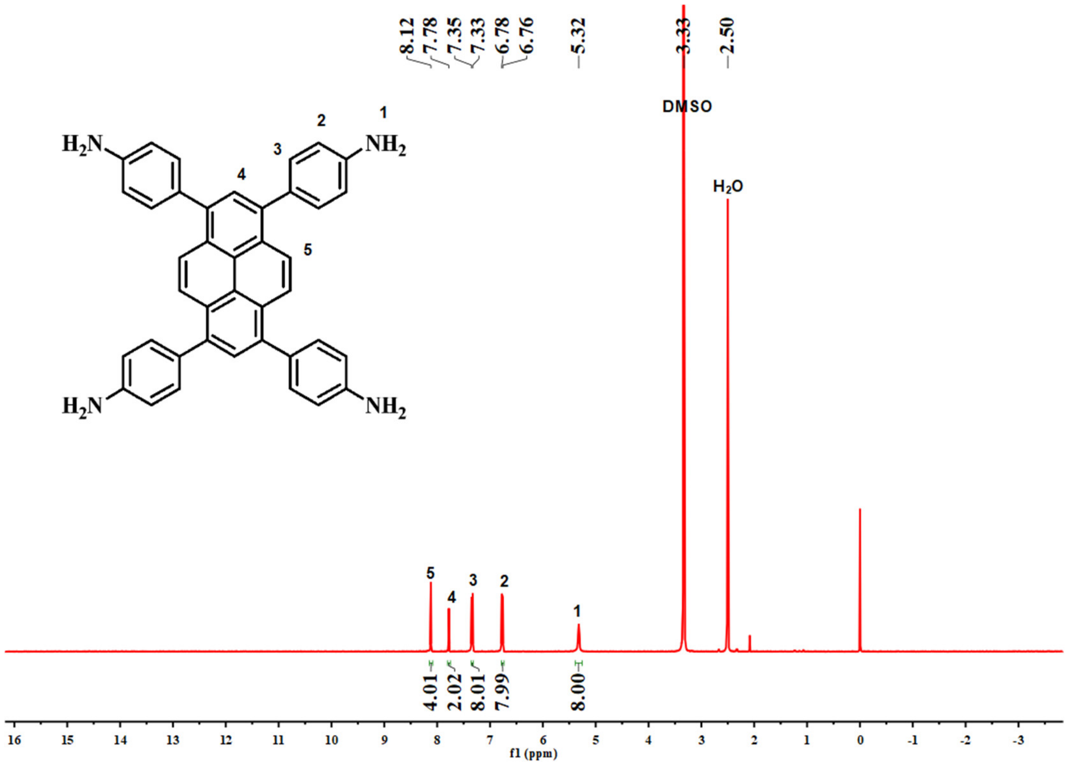

Figure S7. ^{1}H NMR spectrum of PyTTA.

Synthesis of POP-E.

TAPE (19.6 mg) and TFPA (27.6 mg) were first added to a 5 mL Pyrex tube. Then 1.0 mL of 1,2-dichlorobenzene and 0.1 mL of acetic acid were added. Subsequently, the tube was flash frozen at 77 K through liquid nitrogen and degassed by three freeze–pump–thaw cycles. After that, the tube was heated at 120 °C for 72 h. When the reaction was completed, the tube was cooled down to room temperature and the resultant yellow precipitate was purified using Soxhlet extraction with anhydrous tetrahydrofuran for 24 h. At last, the material was vacuum-dried at 120 °C to afford deep yellow POP-E. (30.5 mg, 70% in yield). Theory: C, 88.04; H, 5.54; N, 6.42. Found: C, 82.88; H, 4.82; N, 6.47.

Synthesis of POP-T. This used TTF-4NH_2 (28.5 mg) and TFPA (27.6 mg) but the reaction steps are the same as the synthesis of POP-E. This produced a dark red powder (39.3 mg, 75% in yield). Theory: C, 77.83; H, 4.61; N, 5.34; S, 12.22. Found: C, 77.12; H, 4.72; N, 5.22; S, 11.87.

Synthesis of POP-P. This used PyTTA (28.3 mg) and TFPA (27.6 mg) but the reaction steps are the same as the synthesis of POP-E. This produced a yellow-green powder (40.8 mg, 78% in yield). Theory: C, 89.45; H, 5.20; N, 5.25. Found: C, 87.62; H, 5.40; N, 5.07.

Section 3. Iodine adsorption and desorption

Iodine vapor adsorption experimental procedure:

A certain amount of POPs and excess iodine were placed into two open vials, respectively and then reansfered them into a big chamber. The chamber was sealed tightly and moved to a convection oven (348 K) for iodine vapor adsorption experiment. The weight of the vial that loaded POPs was recorded at different exposure times and the adsorption curves of the samples were thus plotted.

The iodine uptake capacity of POPs was evaluated according to the following equation:

$$\alpha = \frac{(m2 - m1)}{m1}$$

where α is the iodine vapor uptake capacity, and m_1 and m_2 represent the

weight of sample before and after the capture of iodine.

Iodine solution adsorption experiment procedures:

The adsorption experiment of POPs in solution was carried out in n-hexane solution of iodine. A series of iodine-n-hexane solutions (10.0 mL) with different concentrations (100 mg·L^{-1}, 300 mg·L^{-1} and 500 mg·L^{-1}) were first prepared. Then, POPs (5.0 mg) were added to the solution and the system was transferred to dark conditions for adsorption for 72 h. The iodine adsorption behavior in hexane was tested by UV-vis spectroscopy. The iodine uptake capacity of POPs in solution was evaluated according to the following equation:

$$R = \frac{(C0 - Ct)}{C0}$$

where R is the iodine uptake capacity of POPs in hexane solution. C_t represents the iodine concentration of the supernatant after adsorption of iodine and C_0 represents the iodine concentration of the initial supernatant.

Section 4. Characterization of POPs

PXRD spectra

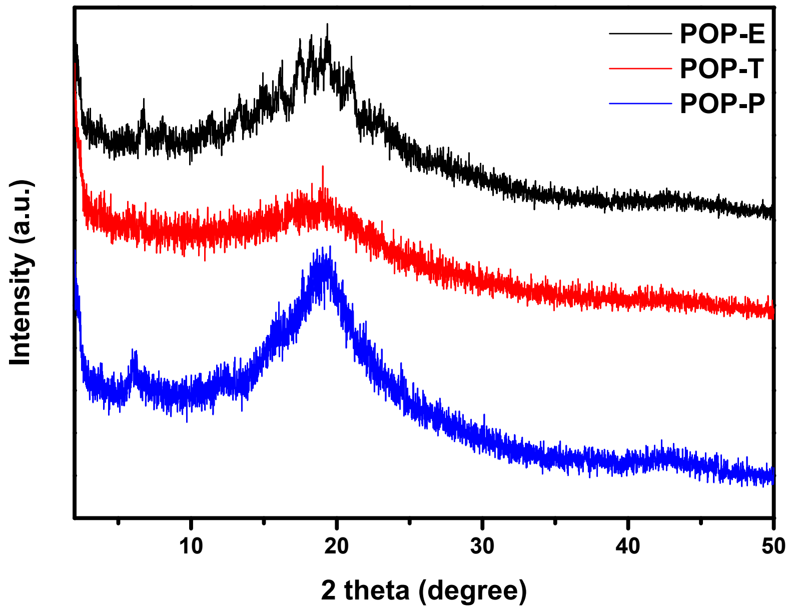

Fig S8 PXRD spectra of POP-E, POP-T and POP-P.

TGA spectra

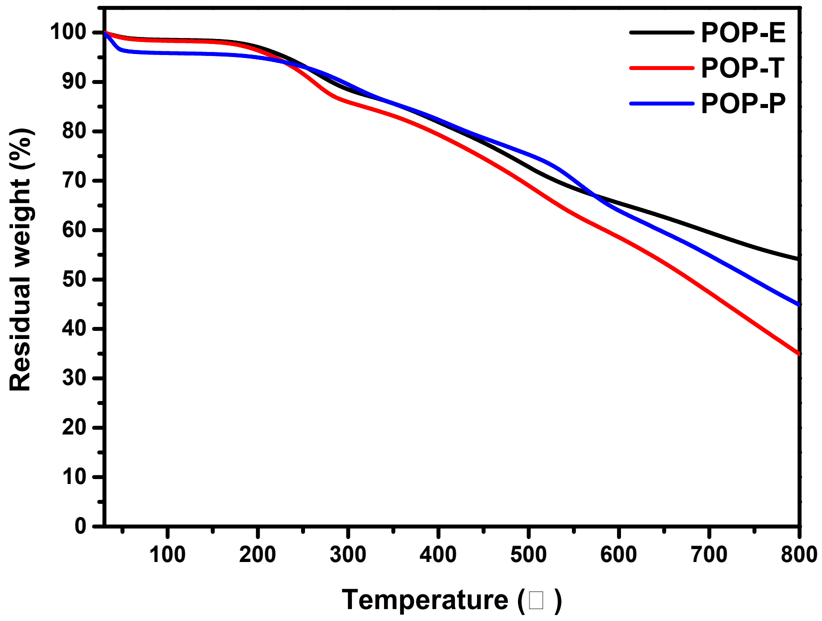

Figure S9. TGA curves of POP-E, POP-T and POP-P.

SEM

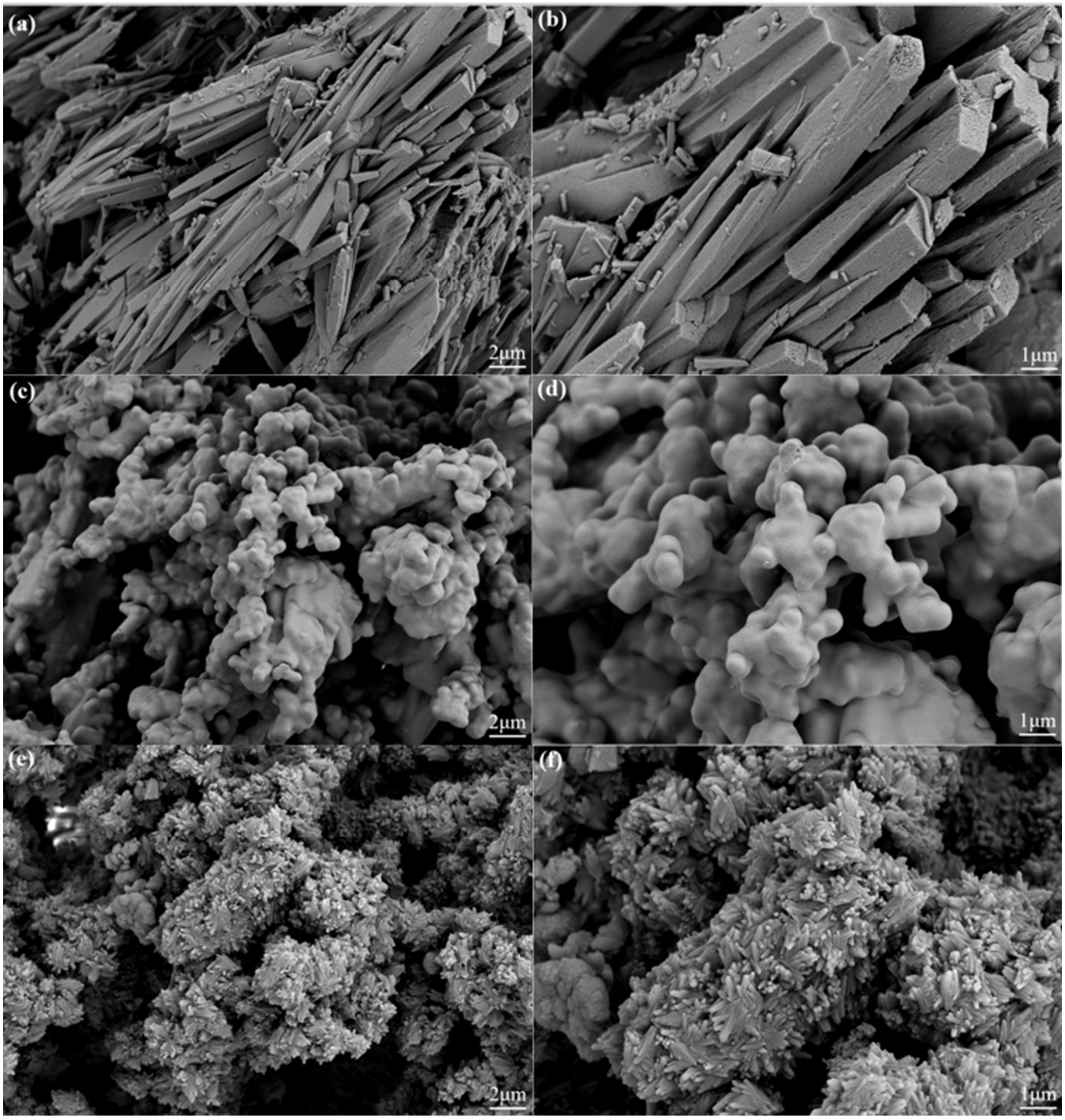

Figure S10. SEM images of (a) and (b) for POP-E; (c) and (d) for POP-T; (e) and (f) for POP-P.

Section 5. Iodine vapor adsorption and desorption

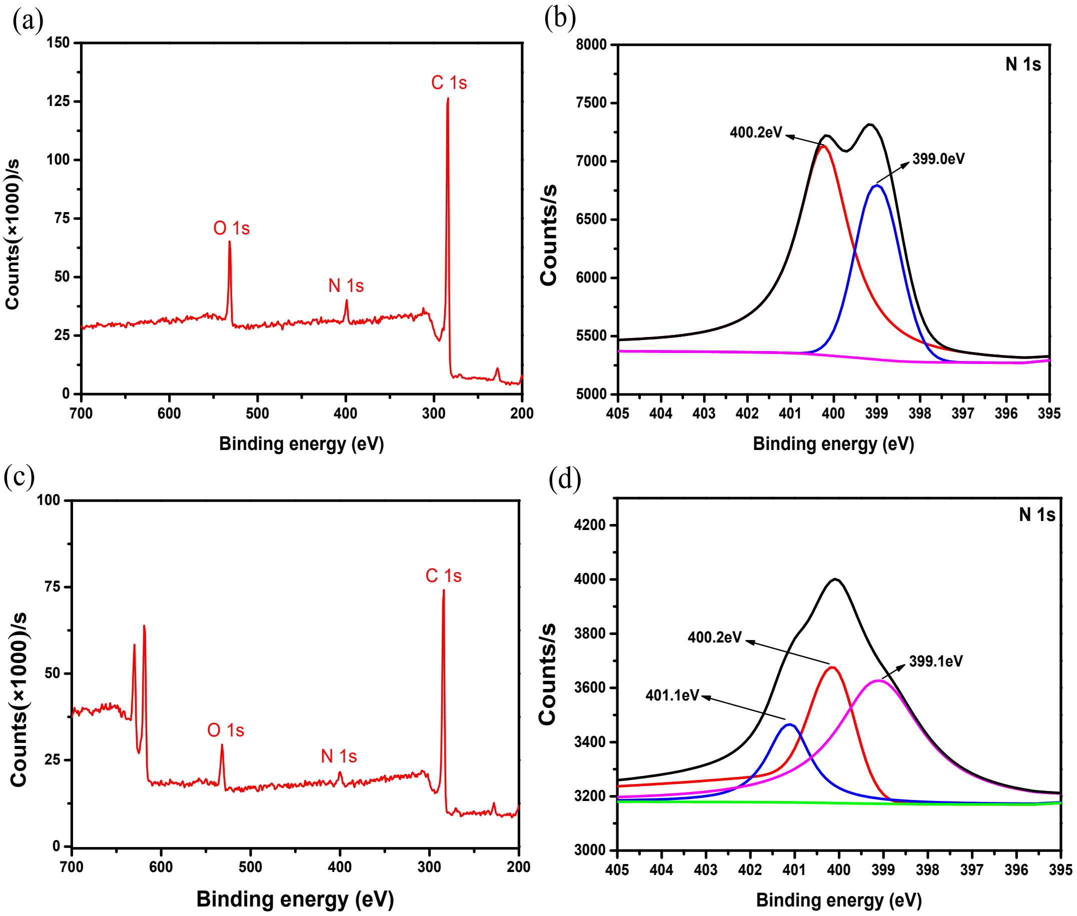

Figure S11. (a) XPS spectrum of POP-T, (b) XPS spectrum of N 1s in POP-T, (c) XPS spectrum of I_2@POP-T, (d) XPS spectrum of N 1s in I_2@POP-T.

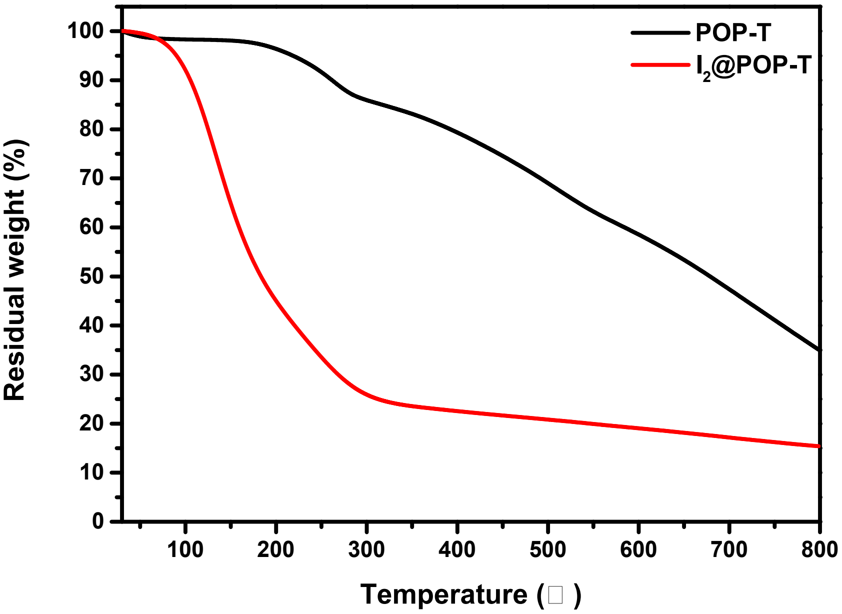

Fig S12 TGA curves of POP-T and I_2@POP-T.

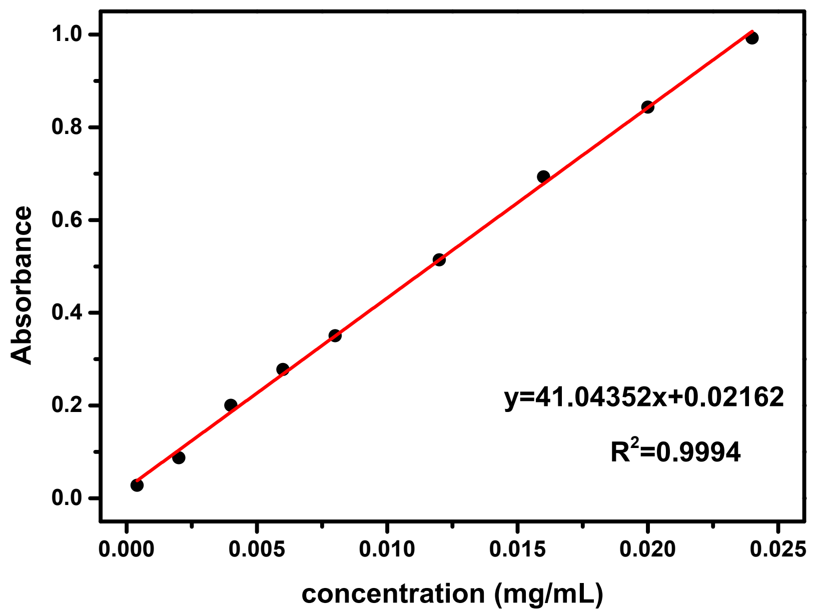

Figure S13. Working curve for iodine in methanol solution obtained from UV-Vis spectroscopy absorbed at 226 nm.

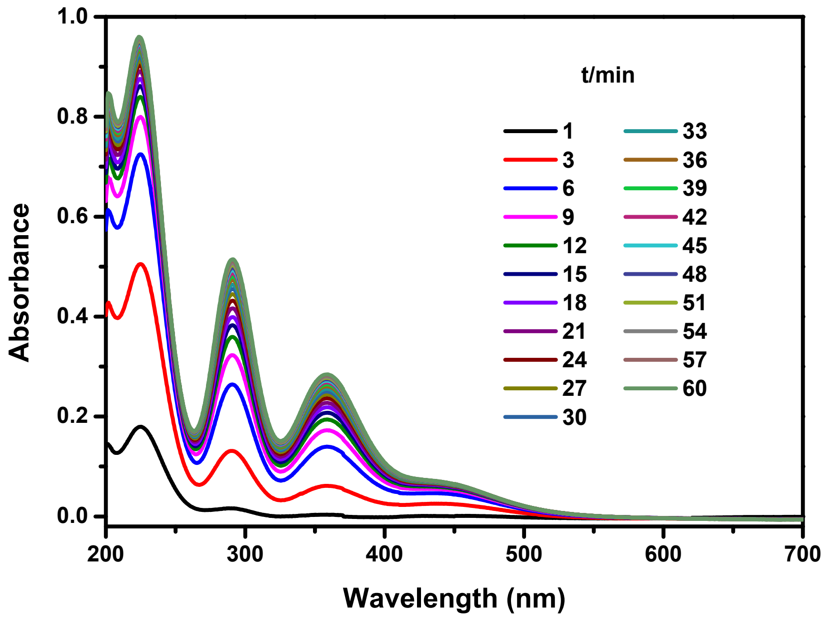

Figure S14. UV-vis spectra for I_2@POP-T (0.1 mg) released from methanol solution (3 mL) at different times.

Table S1. Summary of representatively reported adsorbents with our work for iodine vapor adsorption under ambient pressure.

**Adsorbents**	**S_{BET} ($m^2\ g^{-1}$)**	**Pore volumn ($cm^3\ g^{-1}$)**	**Temperature (°C)**	**Iodine capability ($g\ g^{-1}$)**	**Ref**
JUC-561	2359	1.92	75	8.19	5
TPB-DMTP	1927	1.28	77	6.26	6
TPT-BD-COF	109	0.3	75	5.43	7
JUC-560	1815	1.11	75	5.20	5
TTA-TTB COF	1733	1.01	77	4.95	6
SIOC-COF-7	618	0.41	75	4.81	8
ETTA-TPA COF	1822	0.95	77	4.79	6
COF-DL229	1762	0.64	75	4.7	9
NDB-H	116.9	0.13	75	4.43	10
NDB-S	56.5	0.11	75	4.25	10
**POP-T**	**18.3**	**0.027**	**75**	**3.94**	**This work**
POP-2	41	n.a.	80	3.82	11
TFBCz-PDA	1441	0.74	77	3.7	6
POP-1	12	n.a.	80	3.57	11
Micro-COF-2	1056	0.71	75	3.5	12
**POP-E**	**37**	**0.058**	**75**	**3.49**	**This work**
ADB-HS	148.2	0.14	75	3.45	10
SCMP-II	119.8	n.a.	80	3.45	13
ADB-S	41.5	0.07	75	3.42	10
HCMP-3	82	0.08	85	3.36	14
Meso-COF-4	926	1.01	75	3.3	12
**POP-P**	**27.8**	**0.037**	**75**	**3.27**	**This work**
CalP4_Li	445	0.588	75	3.12	15

HCMP-1	430	0.22	85	2.91	14
AzoPPN	400	0.68	77	2.9	16
Micro-COF-1	816	0.59	75	2.9	12
BDP-CPP-1	635	0.78	75	2.83	17
HCMP-2	153	0.06	85	2.81	14
PAF-24	136	n.a.	75	2.76	18
TTA-TFB COF	1163	0.55	77	2.76	6
PAF-23	82	n.a.	75	2.71	18
PAF-25	262	n.a.	75	2.60	18
CalP3_Li	308	0.558	75	2.48	15
NAPOP-3	702	1.19	75	2.41	19
NAPOP-2	458	0.88	75	2.39	19
Azo-Trip	510.4	0.47	80	2.38	20
BDP-CPP-2	235	0.18	75	2.23	17
HCMP-4	28	n.a.	85	2.22	14
CalP4	759	1.08	75	2.20	15
CMPN-3	1368	2.36	75	2.08	21
NAPOP-1	657	1.74	75	2.06	19
NiP-CMP	2630	2.23	80	2.02	22
NTP	1067	n.a.	75	1.80	24
3D-PPy	16	n.a.	80	1.6	25
Por-Py-CMP	1041	0.81	77	1.3	26
COTs	35	n.a.	40	1.2	27
CMPN-2	339	0.39	75	1.1	21
CMPN-1	230	0.14	75	0.97	21
CF / COF	166	n.a.	77	0.82	28
JUC-Z2	2081	1.45	60	0.80	23
PAF-1	5600	2.63	60	0.74	23
PPy	6	n.a.	80	0.63	25
COFs@cotton	124	n.a.	77	0.53	29

Section 6. Iodine solution adsorption experiment

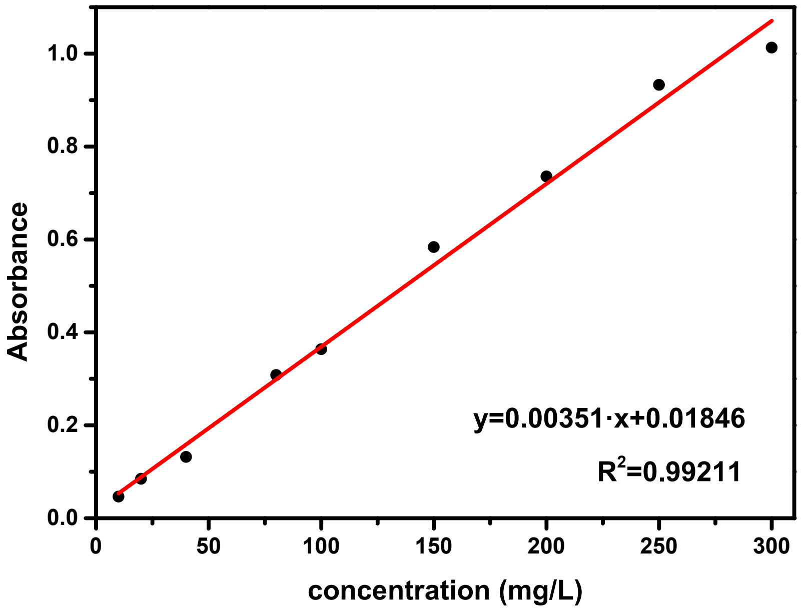

Figure S15. Working curve for iodine n-hexane solution with various concentrations obtained from UV-vis spectroscopy absorbed at 523 nm.

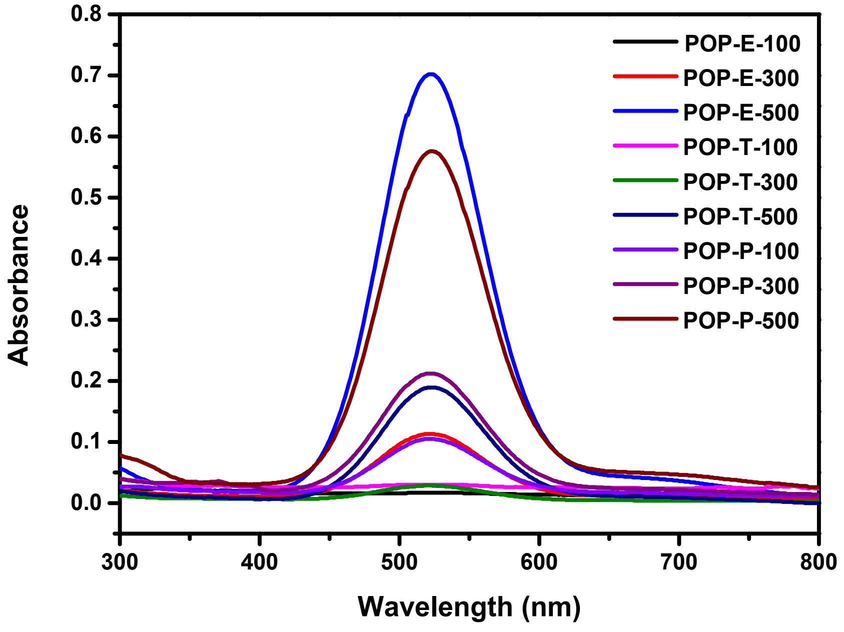

Figure S16. UV-vis spectra for POP-E, POP-T and POP-P (5.0 mg) at different concentrations of iodine in n-hexane solution (100 $mg\cdot L^{-1}$, 300 $mg\cdot L^{-1}$ and 500 $mg\cdot L^{-1}$, 10mL) after the POPs were adsorbed under dark conditions for 72 h.

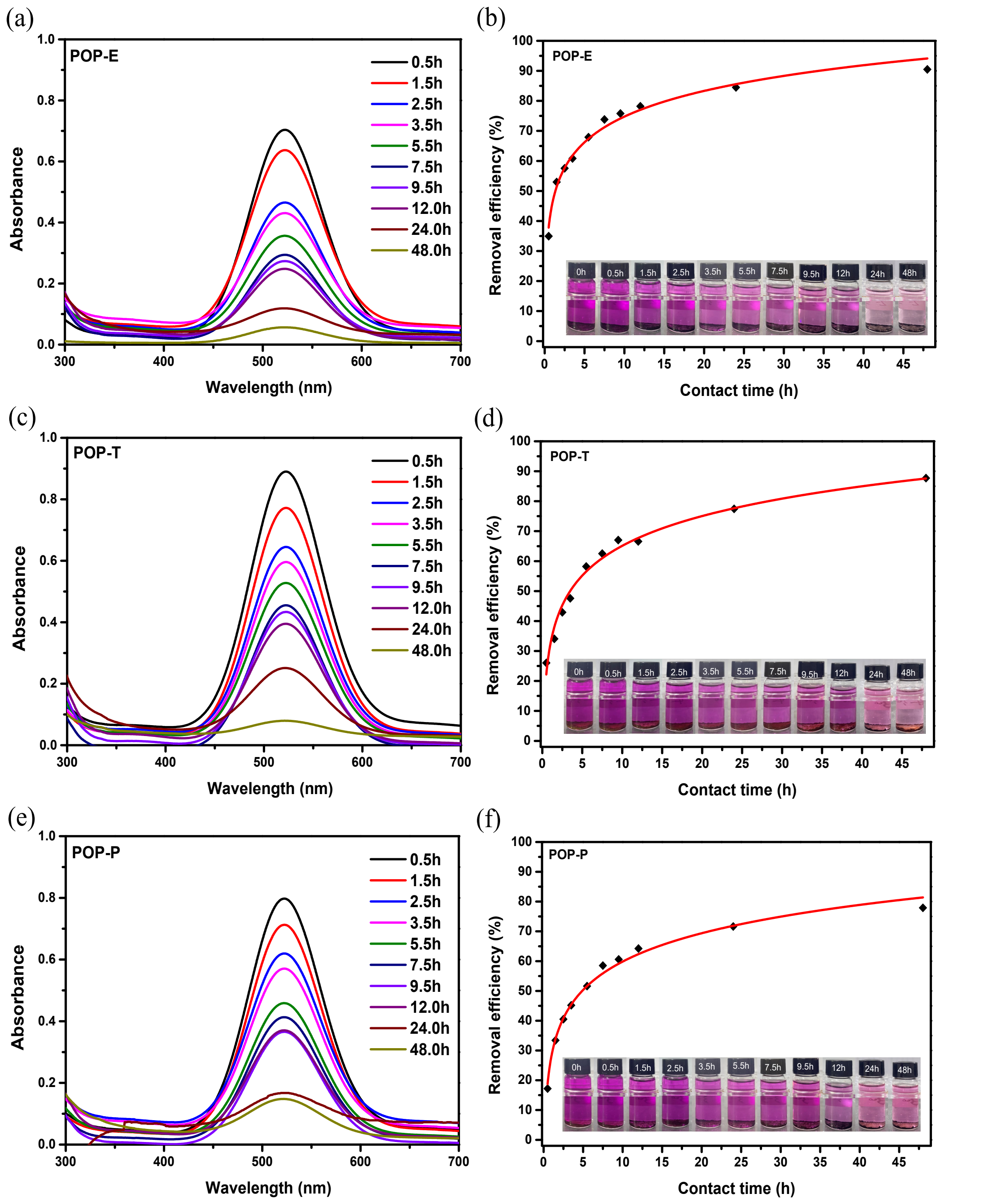

Figure S17. (a), (c) and (e) UV-vis spectra for POP-E, POP-T and POP-P (5.0 mg) in 300 $mg\cdot L^{-1}$ of iodine in n-hexane solution (10 mL) at different times. (b), (d) and (f) Fitting curves of adsorption of POP-E, POP-T and POP-P in 300 $mg\cdot L^{-1}$ iodine in n-hexane solution.

References:

[1] Plietzsch, O.; Schilling, C. I.; Tolev, M.; Nieger, M.; Richert, C.; Muller, T.; Bräse, S., Four-fold click reactions: Generation of tetrahedral methane- and adamantane-based building blocks for higher-order molecular assemblies. *Organic & Biomolecular Chemistry* **2009,** *7* (22), 4734-4743.

[2] Chang, J.; Li, H.; Zhao, J.; Guan, X.; Li, C.; Yu, G.; Valtchev, V.; Yan, Y.; Qiu, S.; Fang, Q., Tetrathiafulvalene-based covalent organic frameworks for ultrahigh iodine capture. *Chemical Science* **2021,** *12* (24), 8452-8457.

[3] Li, G.; Zhang, B.; Yan, J.; Wang, Z., Micro- and mesoporous poly(Schiff-base)s constructed from different building blocks and their adsorption behaviors towards organic vapors and CO2 gas. *Journal of Materials Chemistry A* **2014,** *2* (44), 18881-18888.

[4] Lu, J.; Zhang, J., Facile synthesis of azo-linked porous organic frameworks via reductive homocoupling for selective CO_2 capture. *Journal of Materials Chemistry A* **2014,** *2* (34), 13831-13834.

[5] Ascherl, L.; Evans, E. W.; Gorman, J.; Orsborne, S.; Bessinger, D.; Bein, T.; Friend, R. H.; Auras, F., Perylene-Based Covalent Organic Frameworks for Acid Vapor Sensing. *Journal of the American Chemical Society* **2019,** *141* (39), 15693-15699.

[6] Wang, P.; Xu, Q.; Li, Z.; Jiang, W.; Jiang, Q.; Jiang, D., Exceptional Iodine Capture in 2D Covalent Organic Frameworks. *Advanced Materials* **2018,** *30* (29), 1801991.

[7] Guo, X.; Tian, Y.; Zhang, M.; Li, Y.; Wen, R.; Li, X.; Li, X.; Xue, Y.; Ma, L.; Xia, C.; Li, S., Mechanistic Insight into Hydrogen-Bond-Controlled Crystallinity and Adsorption Property of Covalent Organic Frameworks from Flexible Building Blocks. *Chemistry of Materials* **2018,** *30* (7), 2299-2308.

[8] Yin, Z.-J.; Xu, S.-Q.; Zhan, T.-G.; Qi, Q.-Y.; Wu, Z.-Q.; Zhao, X., Ultrahigh volatile iodine uptake by hollow microspheres formed from a heteropore covalent organic framework. *Chemical Communications* **2017,** *53* (53), 7266-7269.

[9] Wang, C.; Wang, Y.; Ge, R.; Song, X.; Xing, X.; Jiang, Q.; Lu, H.; Hao, C.; Guo, X.; Gao, Y.; Jiang, D., A 3D Covalent Organic Framework with Exceptionally High Iodine Capture Capability. *Chemistry-a European Journal* **2018,** *24* (3), 585-589.

[10] Guo, Z.; Sun, P.; Zhang, X.; Lin, J.; Shi, T.; Liu, S.; Sun, A.; Li, Z., Amorphous Porous Organic Polymers Based on Schiff-Base Chemistry for Highly Efficient Iodine Capture. *Chemistry – An Asian Journal* **2018,** *13* (16), 2046-2053.

[11] Qian, X.; Wang, B.; Zhu, Z.-Q.; Sun, H.-X.; Ren, F.; Mu, P.; Ma, C.; Liang, W.-D.; Li, A., Novel N-rich porous organic polymers with extremely high uptake for capture and reversible storage of volatile iodine. *Journal of Hazardous Materials* **2017,** *338*, 224-232.

[12] An, S.; Zhu, X.; He, Y.; Yang, L.; Wang, H.; Jin, S.; Hu, J.; Liu, H.,

Porosity Modulation in Two-Dimensional Covalent Organic Frameworks Leads to Enhanced Iodine Adsorption Performance. *Industrial & Engineering Chemistry Research* **2019,** *58* (24), 10495-10502..

[13] Ren, F.; Zhu, Z.; Qian, X.; Liang, W.; Mu, P.; Sun, H.; Liu, J.; Li, A., Novel thiophene-bearing conjugated microporous polymer honeycomb-like porous spheres with ultrahigh iodine uptake. *Chemical Communications* **2016,** *52* (63), 9797-9800.

[14] Liao, Y.; Weber, J.; Mills, B. M.; Ren, Z.; Faul, C. F. J., Highly Efficient and Reversible Iodine Capture in Hexaphenylbenzene-Based Conjugated Microporous Polymers. *Macromolecules* **2016,** *49* (17), 6322-6333.

[15] Shetty, D.; Raya, J.; Han, D. S.; Asfari, Z.; Olsen, J.-C.; Trabolsi, A., Lithiated Polycalix[4]arenes for Efficient Adsorption of Iodine from Solution and Vapor Phases. *Chemistry of materials* **2017,** *29* (21), 8968-8972.

[16] Li, H.; Ding, X.; Han, B.-H., Porous Azo-Bridged Porphyrin–Phthalocyanine Network with High Iodine Capture Capability. *Chemistry – A European Journal* **2016,** *22* (33), 11863-11868.

[17] Zhu, Y.; Ji, Y.-J.; Wang, D.-G.; Zhang, Y.; Tang, H.; Jia, X.-R.; Song, M.; Yu, G.; Kuang, G.-C., BODIPY-based conjugated porous polymers for highly efficient volatile iodine capture. *Journal of Materials Chemistry A* **2017,** *5* (14), 6622-6629.

[18] Yan, Z.; Yuan, Y.; Tian, Y.; Zhang, D.; Zhu, G., Highly Efficient Enrichment of Volatile Iodine by Charged Porous Aromatic Frameworks

with Three Sorption Sites. *Angewandte Chemie International Edition* **2015,** *54* (43), 12733-12737.

[19] Weng, J.-Y.; Xu, Y.-L.; Song, W.-C.; Zhang, Y.-H. Tuning the adsorption and fluorescence properties of aminal-linked porous organic polymers through N-heterocyclic group decoration. Journal of Polymer Science Part A: Polymer Chemistry 2016, 54 (12), 1724.

[20] Dang, Q.-Q.; Wang, X.-M.; Zhan, Y.-F.; Zhang, X.-M., An azo-linked porous triptycene network as an absorbent for CO_2 and iodine uptake. *Polymer Chemistry* **2016,** *7* (3), 643-647.

[21] Chen, Y.; Sun, H.; Yang, R.; Wang, T.; Pei, C.; Xiang, Z.; Zhu, Z.; Liang, W.; Li, A.; Deng, W., Synthesis of conjugated microporous polymer nanotubes with large surface areas as absorbents for iodine and CO_2 uptake. *Journal of Materials Chemistry A* **2015,** *3* (1), 87-91.

[22] A, S.; Zhang, Y.; Li, Z.; Xia, H.; Xue, M.; Liu, X.; Mu, Y., Highly efficient and reversible iodine capture using a metalloporphyrin-based conjugated microporous polymer. *Chemical Communications* **2014,** *50* (62), 8495-8498.

[23] Pei, C.; Ben, T.; Xu, S.; Qiu, S., Ultrahigh iodine adsorption in porous organic frameworks. *Journal of Materials Chemistry A* **2014,** *2* (20), 7179-7187.

[24] Ma, H.; Chen, J.-J.; Tan, L.; Bu, J.-H.; Zhu, Y.; Tan, B.; Zhang, C., Nitrogen-Rich Triptycene-Based Porous Polymer for Gas Storage and

Iodine Enrichment. *ACS Macro Letters* **2016,** *5* (9), 1039-1043.

[25] Mu, P.; Sun, H.; Chen, T.; Zhang, W.; Zhu, Z.; Liang, W.; Li, A., A Sponge-Like 3D-PPy Monolithic Material for Reversible Adsorption of Radioactive Iodine. *Macromolecular Materials and Engineering* **2017,** *302* (10).

[26] Park, K. C.; Cho, J.; Lee, C. Y., Porphyrin and pyrene-based conjugated microporous polymer for efficient sequestration of CO2 and iodine and photosensitization for singlet oxygen generation. *RSC Advances* **2016,** *6* (79), 75478-75481.

[27] Das, G.; Skorjanc, T.; Sharma, S. K.; Prakasam, T.; Platas-Iglesias, C.; Han, D. S.; Raya, J.; Olsen, J.-C.; Jagannathan, R.; Trabolsi, A., Morphological Diversity in Nanoporous Covalent Organic Materials Derived from Viologen and Pyrene. *Chemnanomat* **2018,** *4* (1), 61-65.

[28] Li, L.; Chen, R.; Li, Y.; Xiong, T.; Li, Y., Novel cotton fiber-covalent organic framework hybrid monolith for reversible capture of iodine. *Cellulose* **2020,** *27* (10), 5879-5892.

[29] Li, Y.; Li, Y.; Zhao, Q.; Li, L.; Chen, R.; He, C., Cotton fiber functionalized with 2D covalent organic frameworks for iodine capture. *Cellulose* **2020,** *27* (3), 1517-1529.
